# The geobiological nitrogen cycle: From microbes to the mantle

**DOI:** 10.1111/gbi.12228

**Published:** 2017-02-03

**Authors:** A. L. Zerkle, S. Mikhail

**Affiliations:** ^1^ School of Earth & Environmental Sciences and Centre for Exoplanet ScienceUniversity of St AndrewsSt AndrewsFifeUK

## Abstract

Nitrogen forms an integral part of the main building blocks of life, including DNA, RNA, and proteins. N_2_ is the dominant gas in Earth's atmosphere, and nitrogen is stored in all of Earth's geological reservoirs, including the crust, the mantle, and the core. As such, nitrogen geochemistry is fundamental to the evolution of planet Earth and the life it supports. Despite the importance of nitrogen in the Earth system, large gaps remain in our knowledge of how the surface and deep nitrogen cycles have evolved over geologic time. Here, we discuss the current understanding (or lack thereof) for how the unique interaction of biological innovation, geodynamics, and mantle petrology has acted to regulate Earth's nitrogen cycle over geologic timescales. In particular, we explore how temporal variations in the external (biosphere and atmosphere) and internal (crust and mantle) nitrogen cycles could have regulated atmospheric *p*N_2_. We consider three potential scenarios for the evolution of the geobiological nitrogen cycle over Earth's history: two in which atmospheric *p*N_2_ has changed unidirectionally (increased or decreased) over geologic time and one in which *p*N_2_ could have taken a dramatic deflection following the Great Oxidation Event. It is impossible to discriminate between these scenarios with the currently available models and datasets. However, we are optimistic that this problem can be solved, following a sustained, open‐minded, and multidisciplinary effort between surface and deep Earth communities.

## 1 Introduction

Understanding the nitrogen cycle is part of a dynamic geobiological puzzle, for which the ultimate goal is illuminating the mechanics responsible for the development of habitability on Earth and on other planets, in our solar system and beyond. For example, nitrogen is a key element in the structure of amino acids, proteins, nucleic acids, and other molecules vital to life and is also the dominant component of Earth's atmosphere. In addition, the oxidation of ammonic nitrogen (NH_4_^+^) in the mantle has been proposed as a source of liquid water to the early Earth (Li & Keppler, 2014), and N_2_ could have played an important role in maintaining Earth's surface above the freezing point of water in the presence of the faint young sun (e.g., Goldblatt et al., 2009).

The chemistry of Earth's atmosphere is in a constant state of disequilibrium due to atmospheric photochemistry, life's collective metabolisms, chemical weathering, and the large‐scale geochemical fluxes imposed by plate tectonics and related volcanism (Bebout, Fogel, & Cartigny, 2013). However, atmospheric chemistry is not a constant though time (Barry & Hilton, 2016; Mikhail & Sverjensky, 2014). The most dramatic geochemical transition since the formation of the atmosphere was biological rather than geological in nature. Sometime in the mid‐ to late Archean (e.g., Farquhar, Zerkle, & Bekker, 2011), microbial life developed the ability to perform oxygenic photosynthesis, which uses energy from the sun and raw materials extracted from the geosphere (CO_2_ + H_2_O) to generate energy, construct essential building materials, and releases oxygen in a gas phase (O_2_) as a waste product. Over time this biological revelation cumulatively oxygenated Earth's surface. The result is that Earth's atmosphere became highly reactive, unlike the atmospheres of Mars and Venus which are still dominated by unreactive gases (CO_2_ + N_2_). The combination of a uniquely reactive‐gas‐rich atmosphere and hydrosphere, coupled with subduction zone plate tectonics, means that Earth injects oxidizing material into a relatively reduced mantle (Frost & Mccammon, 2008; Kelley & Cottrell, 2009). This phenomenon is seemingly unique because such biological and tectonic processes are only known to occur on Earth. Here, we compare and contrast current ideas for how this unique interaction of biological innovation, geodynamics, and mantle petrology could have acted to regulate the geobiological nitrogen cycle over Earth history.

## 2 Background

### 2.1 Surficial nitrogen cycle

**Figure 1:**
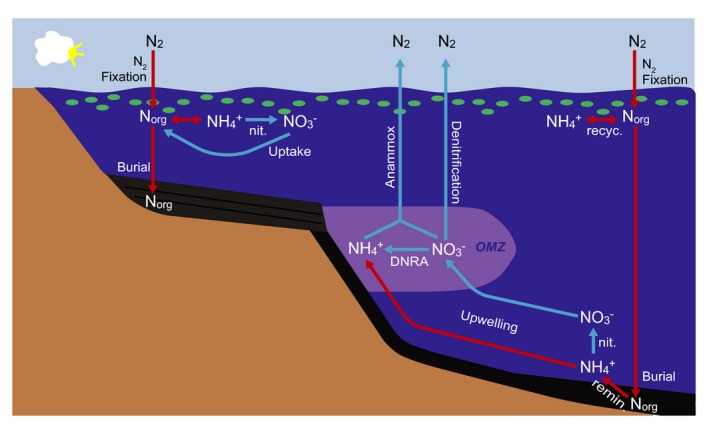
Basin‐scale model of the modern marine biogeochemical nitrogen cycle. The red pathways are anaerobic pathways that would have presumably dominated in anoxic Archean environments. The blue pathways require oxygen (or an oxidized nitrogen species) and would presumably have proliferated after the GOE. Note that for simplicity the nitrite and nitrate pools are shown combined, as NO_3_^−^. Additional abbreviations are as follows: OMZ = oxygen minimum zone (shown in purple); DNRA = dissimilatory nitrate reduction to ammonium; nit. = nitrification; remin. = remineralization; recyc. = recycling

Molecular nitrogen in the gas‐phase (N_2_) is the largest surficial reservoir of nitrogen and comprises around 78% of Earth's atmosphere. Atmospheric nitrogen is incorporated into the biosphere via the process of N_2_ fixation, whereby specialized prokaryotes convert this inert N_2_ gas into biomolecules (as C‐NH_2_) (Figure 1). Nitrogen fixation is an energetically expensive process which requires 16 ATP to break the triply bonded N_2_ molecule. N_2_‐fixing organisms (termed “diazotrophs”) accomplish this feat by utilizing the nitrogenase metallo‐enzyme complex, which contains Fe and Mo in its most efficient form.

Nitrogen fixed as ammonium (NH_4_^+^) is dominantly recycled by the biosphere or sequentially oxidized to nitrite (NO_2_^−^) and nitrate (NO_3_^−^). Ammonium oxidation (“nitrification”) generally requires molecular oxygen (Carlucci & Mcnally, 1969). These “oxidized” nitrogen compounds provide an important nutrient source for eukaryotic primary producers. Alternatively, nitrate (and some nitrite) can also be reduced back to ammonium via dissimilatory nitrate reduction to ammonium (DNRA), another process that was described fairly recently in the modern system (Lam et al., 2009).

Fixed nitrogen can be returned to the atmosphere as N_2_ via several chemotrophic metabolisms, including denitrification (the reduction of NO_3_^−^ to N_2_) and anaerobic ammonium oxidation (anammox, the oxidation of NH_4_^+^ to N_2_ using NO_2_^−^; Dalsgaard, Thamdrup, & Canfield, 2005). These processes, which result in the loss of fixed N from the biosphere, occur in soils, marine sediments, and oxygen minimum zones, completing the biological nitrogen cycle (Figure 1).

All life requires N in a reduced form, which can either be taken up directly as ammonium or enzymatically reduced during nitrate assimilation. Once assimilated into biomass, organic nitrogen is recycled via release and re‐assimilation of organic N within the surface ocean, or subsequently regenerated during organic matter remineralization in sediments. Over geologic timescales, some small amount of fixed nitrogen can leak out of the biosphere–ocean system and be buried in the sediments, as organic nitrogen or as remineralized ammonium incorporated into clays during diagenesis (Schroeder & McLain, 1998). During burial, nitrogen‐bearing rocks can undergo metamorphism, which can return a significant fraction of the nitrogen back to the atmosphere (e.g., Haendel, Mühle, Nitzsche, Stiehl, & Wand, 1986). The remainder of N entrained within sediments can be subducted, along with nitrogen sequestered into altered oceanic lithosphere as ammonium and organic species (Busigny, Cartigny, & Philippot, 2011; Halama, Bebout, John, & Scambelluri, 2014). This subduction of sediment and altered oceanic lithosphere constitutes the primary flux of surficial N into the deep Earth.

### 2.2 Deep earth nitrogen cycle

**Figure 2:**
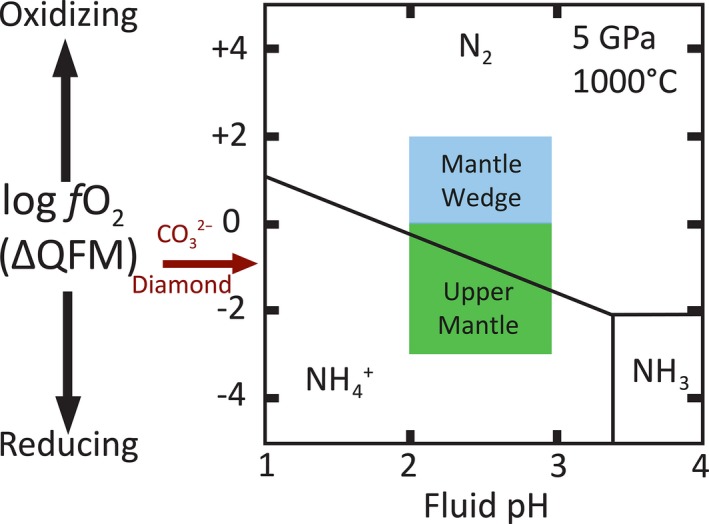
The speciation of aqueous nitrogen at 5 GPa at 1000°C, calculated in equilibrium with Forsterite and Fayalite using the Deep Earth Water model (from Mikhail & Sverjensky, 2014)

Once subducted into the deep Earth, nitrogen has historically been considered an unreactive gas in the field of mantle geochemistry, owing to the strength of the covalent triple N‐bond, and abundance of N_2_ in the atmosphere (e.g., Boyd & Pillinger, 1994; Javoy, Pineau, & Delorme, 1986). Consequently, the behavior of nitrogen has traditionally been assumed to be akin to the unreactive noble gases (e.g., Bergin, Blake, Ciesla, Hirschmann, & Li, 2015; Halliday, 2013; Marty, 1995, 2012; Mikhail, Dobosi, Verchovsky, Kurat, & Jones, 2013). However, empirical data show a diverse variety of nitrogen species are present in the deep Earth, including molecular N_2_, ammonium, diamond (NC_3_), metallic nitride (Fe_3_N, TiN, BN), and nitro‐carbide (for comprehensive reviews of nitrogen in the deep Earth, see Bebout, Lazzeri, & Geiger, 2016; and Johnson & Goldblatt, 2015). All of the aforementioned species of nitrogen behave differently, where their behavior can be described as compatible or incompatible to a host mineral phase, depending on charge and radius of the ion and the occupation site in the mineral (Goldschmidt, 1924). For nitrogen in the mantle, recent experimentally and theoretically determined equilibrium constants have shown that ammonium should dominate over molecular nitrogen in aqueous fluids in the uppermost mantle where the redox state (*f*O_2_) is buffered below the quartz–fayalite–magnetite buffer (QFM) (Li & Keppler, 2014; Mikhail & Sverjensky, 2014) (Figure 2). This is a significant insight, because for nitrogen, compatibility is directly related to speciation: Molecular nitrogen (N_2_^0^) is highly incompatible, whereas ammonium (NH_4_^+^) is likely compatible in potassium‐bearing mineral phases (Watenphul, Wunder, & Heinrich, 2009) and can dissolve as a trace component in potassium‐absent phases (Li, Wiedenbeck, Shcheka, & Keppler, 2013; Watenphul, Wunder, Wirth, & Heinrich, 2010). This is a crucial distinction from the perspective of the global nitrogen cycle, because nitrogen in the molecular form is likely to be outgassed, while ammonium could be stored in mineral phases in the deep Earth (Mikhail & Sverjensky, 2014). We explore this concept, and its potential response to changes in global redox, in the discussion below.

Because Earth is a dynamic planet with active plate tectonics, what goes into the mantle sometimes comes back out, and vice versa. Therefore, the exchange of nitrogen between the surface and interior is governed by subduction (in‐gassing) and volcanism (outgassing), and this interplay ultimately controls atmospheric N_2_ levels (Cartigny & Ader, 2003; Dauphas & Marty, 2004; Kerrich & Jia, 2004; Marty & Dauphas, 2003a,b). However, the further back one looks in time the less data are available, and there is a predictable dearth of data to constrain either the fluxes of nitrogen over geological time, or the partial pressure of atmospheric nitrogen in the deep past. This means we must rely on sparse data and predictive thermodynamic models to estimate past N_2_ dynamics.

## 3 Discussion

### 3.1 Evolution of the biogeochemical N cycle

The emergence and evolution of biogeochemical nitrogen cycling over Earth history remains a major question mark in the field of geobiology (e.g., see recent review by Stüeken, Kipp, Koehler, & Buick, 2016). Abiotic N_2_ reduction to ammonium within hydrothermal systems could have supplied fixed nitrogen to an early deep biosphere (Brandes et al., 1998; Nishizawa, Miyazaki, Makabe, Koba, & Takai, 2014). Once anoxygenic photosynthesis evolved sometime in the Archean, primary productivity would have been most abundant in the surface waters. Dry deposition or fixation by lightning could have provided an abiotic supply of nitrogen to the surface oceans from the atmosphere (Navarro‐Gonzalez, Mckay, & Mvondo, 2001). However, estimates of a very limited supply of nitrogen from these abiotic sources suggest that N_2_ fixation must have evolved very early in life's history to support an ever‐expanding biosphere (Haqq‐Misra, Domagal‐Goldman, Kasting, & Kasting, 2008; Kasting & Siefert, 2001; Kharecha, Kasting, & Siefert, 2005). Phylogenetic reconstructions of genes for the nitrogenase enzyme support its early emergence (Boyd, Hamilton, & Peters, 2011; Fani, Gallo, & Lio, 2000; Raymond, Siefert, Staples, & Blankenship, 2004; Weiss et al., 2016), possibly in an anoxic environment (Boyd & Peters, 2013). In addition, the sedimentary δ^15^N record suggests this process could have been active as far back as ~3.2 Ga (Stüeken, Buick, Guy, & Koehler, 2015). Once N_2_ fixation was established, it is generally assumed that the input of nitrogen to the biosphere could keep pace with primary productivity as long as no other nutrient (e.g., phosphorus or bioactive trace metals; Tyrrell, 1999; Anbar & Knoll, 2002) was limiting.

As the speciation and microbial cycling of nitrogen are highly redox‐dependent, the biogeochemical nitrogen cycle would have changed significantly with the progressive oxygenation of Earth surface environments (e.g., Stüeken et al., 2016). Notably, in Earth's Archean oceans before the buildup of significant oxygen, processes involved in the modern N cycle that required molecular oxygen (or the presence of significant quantities of oxidized N compounds) would have been absent. In particular, nitrification, the stepwise oxidation of ammonium to nitrite and nitrate, requires at least some free oxygen (Carlucci & Mcnally, 1969). Thus, throughout the Archean and earliest Paleoproterozoic the nitrogen cycle would likely have consisted of N_2_ fixation followed by burial and regeneration of NH_4_^+^ in sediments (Figure 1).

Following widespread oxygenation of the marine biosphere during the Great Oxidation Event (GOE) between ~2.4 and 2.3 Ga (e.g., reviewed in Farquhar, Zerkle, & Bekker, 2014), the primary N loss pathways of denitrification and anammox proliferated to take advantage of the newly available oxidized N compounds (Zerkle et al., 2017). In the redox‐stratified oceans that developed during this time period (e.g., Poulton, Fralick, & Canfield, 2010; Scott et al., 2011; Zerkle et al., 2017), nitrification of ammonium‐rich deep waters was confined to the oxic–anoxic interface, with denitrification and anammox occurring just below.

The expansion of an aerobic N cycle during the GOE is supported by temporal trends in sedimentary N isotope records, which show a shift toward more positive δ^15^N values similar to modern marine values by ~2.3 Ga (Beaumont & Robert, 1999; Zerkle et al., 2017). Studies of much older sediments (2.5–2.7 Ga) have interpreted transitory increases in δ^15^N as recording a temporary insurgence of oxidative N cycling, potentially related to localized transient increases in marine O_2_ (Garvin, Buick, Anbar, Arnold, & Kaufman, 2009; Godfrey & Falkowski, 2009; Thomazo, Ader, & Philippot, 2011). These studies suggest that the aerobic N pathways could have evolved immediately following oxygenic photosynthesis, to take advantage of the newly available free oxygen in highly productive coastal environments. However, the isotope effect associated with these N loss pathways would only have been expressed once nitrite/nitrate was available at sufficient levels that their quantitative removal from the ocean did not occur. Therefore, the shift toward more positive δ^15^N values by ~2.3 Ga (Zerkle et al., 2017) represents a turning point in the balance of NO_3_^−^ loss to NO_3_^−^ supply, rather than the exact timing of when the oxidative N cycle first evolved.

The persistence of a deep anoxic water column after the emergence of the aerobic N cycle could have had a dramatic effect on the abundance of fixed nitrogen in the world's oceans. Massive losses of fixed nitrogen via denitrification and anammox are predicted to have occurred during periods of deepwater anoxia (Canfield, Rosing, & Bjerrum, 2006; Fennel, Follows, & Falkowski, 2005), potentially limiting the availability of fixed inorganic nitrogen for primary production. These researchers suggest that such nitrogen limitation could have reduced the burial of organic carbon, and the resulting accumulation of atmospheric oxygen, effectively hindering the evolution of eukaryotes, as well as the continued oxygenation of Earth's surface environments. Nitrogen isotope records from the Mesoproterozoic support nitrogen limitation, particularly within open ocean settings (Stüeken, 2013).

The mid‐Proterozoic nitrogen limitation hypothesis is predicated on the assumptions that the majority of primary productivity was occurring via oxygenic photosynthesis in the surface ocean and that nitrogen fixation could not keep pace with the fixed N loss (and the delivery of other nutrients) to fuel this primary productivity. The evolution of oxygenic photosynthesis in the late Archean would certainly have resulted in a marked increase in global rates of primary production (Canfield et al., 2006). Johnston, Wolfe‐Simon, Pearson, and Knoll (2009) point out that anoxygenic photoautotrophs could also have made an important contribution to primary productivity in the redox‐stratified continental margins that that seemingly dominated for much of the Proterozoic (e.g., Poulton et al., 2010; Scott et al., 2011). Primary producers in marginal sulfidic zones could have had direct access to upwelling nutrients, including ammonium, before it reached the redox interface and was lost to nitrification–denitrification and anammox reactions.

Consumption of upwelling ammonium by anoxygenic phototrophs could have further contributed to the fixed nitrogen deficit in surface waters, such that N_2_‐fixing organisms in the photic zone had a competitive advantage. Many researchers have suggested that N_2_ fixation would also have been limited in the Proterozoic oceans, due to drawdown of bioactive trace metals required for nitrogenase (particularly Mo) under widespread sulfidic conditions (Anbar & Knoll, 2002). Metal limitation experiments with modern N_2_‐fixing cyanobacteria suggest that vanishingly little Mo is actually required to support modern rates of diazotrophy (down to ~5 nM; Zerkle, House, Cox, & Canfield, 2006; Glass, Wolfe‐Simon, & Anbar, 2009). Current estimates of molybdenum concentrations in Precambrian oceans are insufficient to resolve this issue at present (Reinhard, Raiswell, Scott, Anbar, & Lyons, 2009; Scott et al., 2008). However, records of molybdenum in shales suggest that marine molybdenum levels rose to near modern levels at the end of the Neoproterozoic with enhanced oxidative weathering during a second rise in atmospheric O_2_ (Sahoo et al., 2012), potentially further enhancing global N_2_ fixation rates.

**Figure 3:**
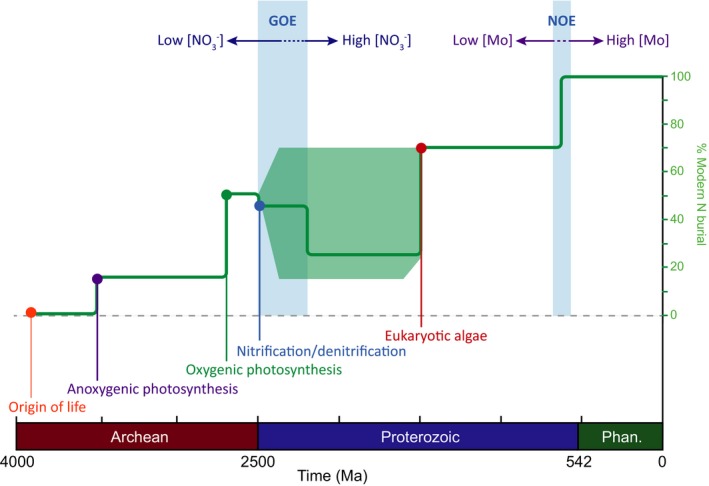
Estimated burial flux of nitrogen through time, following calculations described in the text

As the delivery of nitrogen to the sediments is intimately linked to the biosphere and dependent on redox and other environmental parameters, the burial of nitrogen over geologic time (and thus its flux into the mantle) would have changed in response to these changes in the biogeochemical nitrogen cycle. Using conservative estimates for the timing of major biological and geochemical innovations, we can propose a rough timeline for changes in the burial of N into marine sediments over geologic time (Figure 3). Nitrogen burial is thought to be generally driven by changes in carbon burial over time (e.g., Berner, 2006), which itself plays an important role in regulating Earth surface redox conditions (e.g., Krissansen‐Totton, Buick, & Catling, 2015). For this calculation, we used the productivity estimates of Canfield, Glazer and Falkowsk (2010) for primary production based on an evolutionary progression of anoxygenic photoautotrophy, oxygenic photoautotrophy, and eukaryotic photoautotrophy. We utilized a constant burial rate of 10% total biomass (similar to the modern; Gruber, 2008) with a constant Redfield ratio of 6:1 (C:N). We used denitrification levels estimated from modern marine N balances (e.g., Canfield et al., 2010; Gruber, 2008). The biggest unknown in this calculation is when coupled nitrification/denitrification proliferated and how rates of denitrification responded to changes in ocean chemistry throughout the Proterozoic (as discussed above). For this calculation, we assumed 25% of modern denitrification levels early in the GOE, and an increase to 130% associated with the subsequent buildup of a nitrate reservoir in a largely anoxic ocean, with large error bars to highlight this uncertainty. Finally, we calculated changes in N_2_ fixation rates with enhanced molybdenum delivery during the Neoproterozoic Oxygenation Event (NOE) as a switch from molybdenum‐limited to modern N_2_ fixation rates, based on the experiments of Zerkle et al. (2006). Note that this calculation does not consider any significant loss of nitrogen from the sediments during diagenesis or metamorphic reactions prior to subduction. Notably, this scenario for changes in nitrogen burial over time predicts a progressive increase in the delivery of nitrogen to the deep Earth via subduction (with the exception of the possible mid‐Proterozoic minimum) following an ever‐increasingly efficient biosphere.

### 3.2 The missing nitrogen conundrum

**Figure 4:**
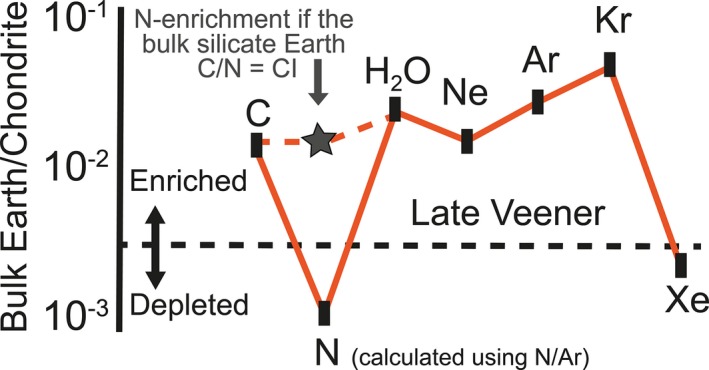
The abundance of volatile elements in the bulk silicate Earth relative to the abundances in carbonaceous chondrites (modified using data from Marty, 2012; and Halliday, 2013)

Our understanding of the evolution of the deep Earth nitrogen cycle and the relevant nitrogen reservoirs is even less well constrained. One puzzle often cited is the “missing nitrogen conundrum,” which follows because the abundance of nitrogen in the bulk silicate Earth (BSE) appears to be significantly lower than that of other volatile elements (Figure 4). The most striking feature of these data is the depletions shown for Xe and N, which could mean that these two elements with contrasting behaviors and masses were both lost early in Earth's history (Bergin et al., 2015) without also depleting Ne, Ar, and Kr—elements that are far more akin to Xe in terms of geochemical behavior, and in the case of Kr, mass. Another explanation is that there exists a hidden nitrogen reservoir in the deep Earth, inferred to be the core or unspecified deep mantle domain (Barry & Hilton, 2016; Halliday, 2013; Marty, 2012). This reservoir is unlikely to be the core because carbon is more siderophile than nitrogen (Dalou, Hirschmann, von der Handt, Mosenfelder, & Armstrong, 2016); therefore, a mantle reservoir is more in line with what is known about the partitioning of carbon and nitrogen during core formation. Furthermore, stable isotope data for diamonds show that nitrogen is subducted back into the deep mantle, implying nitrogen retention during subduction to at least the minimum depth of diamond stability (≥150 km; Mikhail et al., 2014) and possibly into the lower mantle (≥660 km; Palot, Cartigny, Harris, Kaminsky, & Stachel, 2012). This requires a mechanism to transport nitrogen beyond the mantle wedge.

Note that Figure 4 is derived by summing up the assumed abundance of nitrogen in the BSE with the surficial reservoir; however, these values were calculated using N_2_/Ar ratios, which is now known to be inaccurate for the majority of the mantle. In particular, the N_2_/Ar approach underestimates the true volume of the N reservoir, because some (or a lot of) N should exist as lattice‐bound NH_4_^+^ ions (Li & Keppler, 2014; Li et al., 2013; Mikhail & Sverjensky, 2014; Watenphul et al., 2010). Recent experimental data for the partitioning of nitrogen in ferromagnesian minerals show the upper mantle (<250 km depth) can store 20–50 times more nitrogen than the present‐day atmosphere (Li et al., 2013), and preliminary data suggest the transition zone could store even more (Yoshioka, Wiedenbeck, Shcheka, & Keppler, 2016). Therefore, if saturated (which is unlikely), then the upper mantle alone can enable the BSE nitrogen abundance to match the C/N ratio of carbonaceous chondrites (shown on Figure 4).

These datasets and thermodynamic models strongly imply the existence of a deep mantle‐based nitrogen reservoir. Thus, contrary to previous assumptions, it is now clear that the mantle, and not the atmosphere, could form the largest nitrogen reservoir on Earth (Goldblatt et al., 2009; Mikhail & Howell, 2016; Palya, Buick, & Bebout, 2011). Various authors have attempted to provide calculated/assumed reservoir mass fractions for nitrogen in the core, silicate Earth, and the atmosphere (Bebout et al., 2013; Dalou et al. 2016; Goldblatt et al., 2009; Johnson & Goldblatt, 2015; Marty, Zimmermann, Pujol, Burgess, & Philippot, 2013; Palya et al., 2011); however, these estimates all vary significantly, and there is currently no consensus on the relative distributions.

### 3.3 Variations in atmospheric *p*N_2_ over geologic time

Direct data for the partial pressure of atmospheric N_2_ over time are limited, but the majority of available data support lower *p*N_2_ in the Archean compared to the 0.79 bar of N_2_ in the modern atmosphere. For example, Marty et al. (2013) used the N_2_/^36^Ar systematics from fluid inclusions trapped in ~3.0 to 3.5 Ga hydrothermal quartz to suggest the partial pressure of N_2_ of the Archean atmosphere could not exceed 1.1 bar and could be as low as 0.5 bar. More recently, Som et al. (2016) used the size distribution of gas bubbles in basaltic lavas assumed to have solidified at sea level to conclude the atmospheric pressure was 0.23 ± 0.23 (2σ) bar at ~2.7 Ga. In support of these empirical data, the surface‐interior nitrogen flux estimates of Fischer et al. (2002) suggest plate tectonics (in the present) have a net outgassing flux of nitrogen into the atmosphere.

Modeling studies of greenhouse warming on the early Earth have proposed elevated *p*N_2_ in the Archean as a solution to the Faint Young Sun paradox (Airapetian, Glocer, Gronoff, Hebrard, & Danchi, 2016; Goldblatt et al., 2009); however, there is limited evidence to support the suggestion that the Archean had a higher atmospheric *p*N_2_ than the present day. Fossilized raindrop imprints from ~2.7 Ga allow for air density of less than 2 bar, but suggest it was likely less than 1.1 bar (Som, Catling, Harnmeijer, Polivka, & Buick, 2012; but see Kavanagh & Goldblatt, 2015, for a different interpretation). Nishizawa, Sano, Ueno, and Marayama (2007) placed an upper limit of 3.3 times the modern N_2_/^36^Ar on the ~3.5 Ga atmosphere based on fluid inclusions preserved in hydrothermal quartz, but the approach of Nishizawa et al. (2007) provides no lower limit. Therefore, the Archean *p*N_2_ could have been much less than this maximum estimate.

The notion of elevated Archean *p*N_2_ gains some support from indirect evidence, in the form of calculated surface‐interior nitrogen flux estimates for the modern Earth system (Barry & Hilton, 2016; Busigny et al., 2011). These studies argue for plate tectonics in the present having a net in‐gassing flux of nitrogen to the mantle, despite the mantle wedge being too oxidizing to stabilize ammonium (Figure 2). This requires that the subducted nitrogen does not enter the melt or fluid phase and is instead retained in the potassium‐bearing minerals in the downgoing slab. The pathways for this are theoretically well established, whereby NH_4_^+^ is transported into the mantle as a lattice‐bound constituent in potassium‐bearing minerals that vary with increasing depth (Harlow & Davies, 2004). These minerals collectively form a mineralogical conveyor belt for the transfer of nitrogen from the surface to the lowermost mantle (Harlow & Davies, 2004), but the absence of the exchange coefficients for NH_4_^+^/K^+^ for potassium‐bearing minerals means no quantitative assumptions can be made at this stage. Nonetheless, it is plausible that the aforementioned processes would result in the overall surficial nitrogen reservoir being depleted through time, and the mantle abundance of nitrogen would be therefore increasing.

### 3.4 The current state of confusion (aka, Schrodinger's nitrogen cycle)

**Figure 5:**
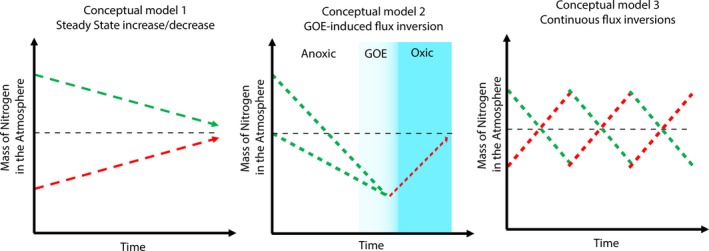
Conceptual models for the mass of nitrogen in Earth's atmosphere through time. The black dashed line represents the present‐day mass of nitrogen in Earth's atmosphere (see text for details). Both time and mass are arbitrary as this is a suite of conceptual models

Given the uncertainties described above, a complete description of the geobiological nitrogen cycle will require solutions for the following outstanding questions: (i) a consensus for the fluxes of nitrogen into and out of the mantle, and how these relate to the surficial nitrogen cycle, and (ii) the validity of the proposed missing nitrogen conundrum. We suggest that there are three plausible scenarios to reconcile these issues within the geobiological nitrogen cycle over Earth's 4.6‐billion‐year history, notably that atmospheric *p*N_2_ has changed unidirectionally (either increased or decreased) over geological time (Figure 5, conceptual model 1), or the direction of the nitrogen abundance of the atmosphere took a dramatic deflection following the Great Oxidation Event (Figure 5, conceptual model 2).

Given the dearth of relevant data, it is impossible to rule out multidirectional changes in *p*N_2_ over geologic time (Figure 5, conceptual model 3), but there is currently no theoretical support or potential mechanism for such variations. Conceptual model 2 is exciting and is mechanistically feasible because of the disparate compatibilities of oxidized and reduced nitrogen, as described above. However, for conceptual model 2 to be true, then the nitrogen cycle has experienced unidirectional change in both directions, with a major turning point at the GOE. In the following, we explore the geobiological implications of these three scenarios.

#### 3.4.1 Scenario #1: Atmospheric pN_2_ has decreased over geologic time

If atmospheric *p*N_2_ was relatively higher in the Archean, this could have resulted in a greater supply of abiotically fixed nitrogen from the atmosphere than previously estimated (Navarro‐Gonzalez et al., 2001). A higher abiotic flux of nitrogen into the biosphere on the early Earth could have allowed for a much later evolution of N_2_ fixation, supporting some phylogenetic reconstructions and molecular clock data (Boyd, Anbar, et al., 2011; Sanchez‐Baracaldo, Ridgwell, & Raven, 2014). A decrease in atmospheric *p*N_2_ over geologic time would also follow from our estimates of enhanced burial of sedimentary nitrogen over geologic timescales (Figure 3). A correlation between nitrogen burial and atmospheric *p*N_2_ would suggest that the biosphere could have directly *driven* changes in *p*N_2_ via the drawdown of nitrogen from the atmosphere, effectively forming an increasingly efficient geobiological pump of nitrogen from the atmosphere into the sediments, for subsequent storage in the deep Earth.

Notably, the scarcity of data available for how these processes, and the dynamic nitrogen cycle, have interacted over geologic time means it is impossible to determine whether the global nitrogen cycle is currently in equilibrium. Therefore, if a progressive drawdown of N_2_ remained unbalanced by mantle degassing (e.g., Busigny et al., 2011), over geologic timescales this geobiological nitrogen pump could be progressively depleting Earth's atmosphere, with currently unexplored consequences for the climate and biogeochemical evolution of a future Earth system.

#### 3.4.2 Scenario #2: Atmospheric pN_2_ has increased over geologic time

On the other hand, a relatively lower *p*N_2_ for the Archean atmosphere is in line with an interplanetary model proposed to explain an enrichment of nitrogen over the primordial noble gases in Earth's atmosphere relative to the atmospheres on the other sampled planets that are devoid of subduction zone plate tectonics (e.g., Mars and Venus; Mikhail & Sverjensky, 2014). At present, subduction zones inject oxidizing material into the mantle wedge above the downgoing slab; at these redox conditions >QFM, thermodynamic calculations predict oxidation of NH_4_ to N_2_ (Figure 2). Because this N_2_ is neutrally charged and highly volatile, it is therefore degassed to the atmosphere. This model predicts Earth's arc systems should degas more nitrogen than mid‐ocean ridge and hot spot volcanism, consistent with observations (e.g., Hilton, Fischer, & Marty, 2002). In addition, the dependence of this N_2_ production on subduction can explain Earth's higher atmospheric N_2_/^36^Ar and N_2_/^20^Ne ratios relative to the Martian and Venusian atmospheres.

Furthermore, if the Archean atmospheric mass was below present levels, then we can explain its increase over time by assuming it must have started with N_2_/^36^Ar and N_2_/^20^Ne ratios similar to Mars and Venus. Mikhail and Sverjensky (2014) applied an empirical nitrogen flux from the Central American volcanic arc system (from Fischer et al., 2002) and amplified it to represent the global flux (factor of 20 or 10, respectively). Following this methodology, the degree of Earth's atmospheric N_2_/^36^Ar and N_2_/^20^Ne enrichment relative to the atmospheres around Mars and Venus can be reproduced over a time period of only 1–2 Ga following the onset of subduction (Mikhail & Sverjensky, 2014). However, we should specify that this time frame more likely reflects the time period from which subduction zones began injecting oxidants, like Fe^3+^ or S^6+^ into the mantle (Kelley & Cottrell, 2009), and not simply the onset of subduction.

This scenario would suggest that an increase in atmospheric *p*N_2_ over geological time was mirrored by an increase in the burial of nitrogen in sediments (Figure 3). A positive coupling implies that the biosphere could have directly *responded to* changes in *p*N_2_ caused by progressive degassing of the mantle. As the atmospheric N_2_ reservoir is orders of magnitude larger than the biosphere, the role of *p*N_2_ in regulating primary productivity over geologic timescales is generally not considered, assuming an infinite supply for biological N_2_ fixation. However, it is unclear how abiotic atmospheric sources of fixed nitrogen (particularly important for early life) could have scaled with *p*N_2_ (Kasting & Siefert, 2001; Navarro‐Gonzalez et al., 2001); if *p*N_2_ was very low in the Archean, this could mean even higher levels of nitrogen stress and an instantaneous requirement for the evolution of N_2_ fixation alongside the early biosphere (Stüeken et al., 2015; Weiss et al., 2016). Furthermore, if N_2_ outgassing increased after the GOE (as discussed below), it could mean that abiotic sources became enhanced in the early Proterozoic, potentially alleviating some of the biological nitrogen stress proposed for the boring billion.

#### 3.4.3 Scenario #3: Atmospheric pN_2_ underwent a major turning point during the GOE

Either in line with or independent of Scenario #2 above, the Great Oxidation Event could have driven a relative increase in *p*N_2_ by altering the speciation and partitioning of nitrogen in subducting sediments. This follows because the speciation and partitioning of nitrogen are redox‐sensitive, as discussed in section 2.2. Therefore, the relative abundance of nitrogen degassed out of the mantle over time must have changed before and after the GOE, because the oxidizing potential of the material subducted (oceanic sediments and altered oceanic crust) must have differed. In particular, post‐GOE the oxidation state of the mantle overlying the slabs would become too oxidizing to stabilize ammonic nitrogen, and molecular nitrogen would be favored (Figure 2). As inert N_2_ molecules are highly volatile and highly incompatible in all mineral phases, this molecular N_2_ would be released as a gas‐phase to the atmosphere, a concept not quantitatively incorporated into recent flux models (Barry & Hilton, 2016; Busigny et al., 2011; Fischer et al., 2002). Therefore, we propose (on a global scale) it is plausible for the flux of nitrogen to have dramatically shifted across the GOE, whereby ingassing dominated pre‐GOE and outgassing dominated post‐GOE. Thus, following the GOE, subduction zones began excessively degassing nitrogen to the atmosphere, which over time resulted in Earth's higher N_2_/^36^Ar and N_2_/^20^Ne ratios relative to the Martian and Venusian atmospheres (Mikhail & Sverjensky, 2014).

A second, as yet unexplored, link between the GOE and atmospheric *p*N_2_ could stem from oxidative weathering. Johnson and Goldblatt (2015) estimated that continental crust could constitute a significant reservoir of nitrogen. This nitrogen is hosted both in sedimentary rocks (as organic matter or NH_4_^+^) and in crystalline rocks (predominantly as NH_4_^+^). There are currently no quantitative estimates we are aware of for the contribution of oxidative weathering to atmospheric N_2_ in the modern Earth system; however, it potentially constitutes an additional N_2_ source that would have been absent or nearly so prior to global oxygenation.

## 4 Conclusions

In summary, subduction zones inject biologically mediated material into the mantle, which is affected by Earth surface redox. This subducted material affects mantle melting and degassing, and can significantly alter Earth's atmospheric chemistry over geologic time. Atmospheric chemistry has been both a driving force, and a response to, biological innovation and evolution. Therefore, the geological and biological nitrogen cycles are intimately linked, albeit with long lag times possibly verging on hundreds to thousands of millions of years.

In the above discussion, we have highlighted some of the looming questions in nitrogen geobiology, and the problems and challenges involved in addressing them. What is painfully obvious from this discussion is that a cross‐disciplinary approach will be crucial in tackling these issues. This requirement goes beyond the typical marriage of low‐temperature geochemistry, microbiology, and sedimentology, toward a deeper connection between biogeochemistry, mantle petrology, and geodynamics. For example, additional thermodynamic and experimental constraints on N speciation under relevant deep Earth conditions are warranted, but these data are only relevant if the input (sourced primarily from biology) is considered. The geobiological nitrogen puzzle will only be solved by considering the biosphere as intimately connected with the geosphere, and *vice versa*. We therefore put this challenge to the *Geobiology* community, to don your hard hats and delve into Earth's interior (accompanied by your hard‐rock colleagues).
